# Latent tuberculosis infection and tuberculosis in children and adolescents

**DOI:** 10.1590/S1679-45082018AO4090

**Published:** 2018-09-10

**Authors:** Cassia Satsuki Ishikawa, Olivia Mari Matsuo, Flavio Sarno

**Affiliations:** 1 Instituto Israelita de Responsabilidade Social, Hospital Israelita Albert Einstein, São Paulo, SP, Brazil; 2 Hospital Israelita Albert Einstein, São Paulo, SP, Brazil

**Keywords:** Tuberculosis, Mycobacterium tuberculosis, Child health services, Child, Child health, Tuberculose, Mycobacterium tuberculosis, Serviços de saúde da criança, Criança, Saúde da criança

## Abstract

**Objective:**

To describe the characteristics of patients diagnosed with tuberculosis and latent tuberculosis infection.

**Methods:**

A retrospective study, between 2012 and 2015, with data from patients of *Programa Einstein na Comunidade de Paraisópolis*. To evaluate possible factors associated with patient's sex and diagnoses of tuberculosis and latent tuberculosis infection, χ^2^ or Fisher's exact tests were used for qualitative variables, and Mann-Whitney test for quantitative or ordinal qualitative variables.

**Results:**

A total of 77 patients were evaluated. Age ranged from 6 months to 13.4 years, with a majority of males (54.5%), aged zero to 4 years (54.5%), diagnosed with latent tuberculosis infection (64.9%), and classified as eutrophic (71.2%). The tuberculin test was positive in 92% and in most cases the values were above 10mm (68.0%). Approximately three-quarters of chest X-ray tests were normal (72.7%). After chest X-ray, computed tomography of thorax was the most ordered exam (29.9%), followed by smear and culture for *Mycobacterium tuberculosis* in the gastric aspirate (28.6%). The frequencies of altered chest X-ray (70.4% *versus* 4.0%), computed tomography of thorax requests (55.6% *versus* 16.0%) and other tests requested (81.5% *versus* 38.0%) were significantly higher in patients with a diagnosis of tuberculosis, relative to those with latent tuberculosis infection, respectively.

**Conclusion:**

In our sample, proportions of altered chest X-ray, and performing computed tomography of thorax and other tests in patients diagnosed with tuberculosis were higher than in those with latent tuberculosis infection.

## RESUMO

**Objetivo:**

Descrever as características de pacientes com diagnóstico de tuberculose e de infecção latente por tuberculose.

**Métodos:**

Estudo retrospectivo, entre os anos de 2012 e 2015, com os dados dos pacientes do ambulatório do Programa Einstein na Comunidade de Paraisópolis. Para se avaliarem possíveis fatores associados ao sexo e aos diagnósticos de tuberculose e infecção latente por tuberculose, foram utilizados os testes χ^2^ ou exato de Fisher, para variáveis qualitativas, e de Mann-Whitney, para variáveis quantitativas ou qualitativas ordinais.

**Resultados:**

Foram avaliados 77 pacientes. A idade variou de 6 meses a 13,4 anos, sendo a maioria do sexo masculino (54,5%), na faixa etária de zero a 4 anos (54,5%), com diagnóstico de infecção latente por tuberculose (64,9%) e categorizada como eutrófica (71,2%). Em 92% dos casos, a prova tuberculínica foi positiva, e a maioria mostrou valores acima de 10mm (68,0%). Cerca de três quartos dos exames de raio X de tórax resultaram normais (72,7%). Depois do raio X de tórax, a tomografia computadorizada de tórax foi o segundo exame mais solicitado (29,9%), seguida da baciloscopia e da cultura do bacilo *Mycobacterium tuberculosis* no aspirado gástrico (28,6%). As frequências de raio X de tórax alterado (70,4% *versus* 4,0%), solicitações de tomografia computadorizada de tórax (55,6% *versus* 16,0%) e outros exames (81,5% *versus* 38,0%) foram significativamente maiores nos pacientes com diagnóstico de tuberculose, em relação àqueles com infecção latente por tuberculose, respectivamente.

**Conclusão:**

Em nossa amostra, as proporções de raio X de tórax alterados e de realização de tomografia computadorizada de tórax e de outros exames nos pacientes com diagnóstico de tuberculose foram maiores em relação àqueles com infecção latente por tuberculose.

## INTRODUCTION

Tuberculosis (TB) is an infectious disease caused by the bacillus ***Mycobacterium tuberculosis*** (BMT), which affects mainly the lungs, but may also affect other organs and tissues. Globally, it is estimated that 1.7 billion people are infected by the bacillus, but only 5 to 15% of these individuals will develop the disease. In 2016, there were 10.4 million new cases of TB, one million of which were among children.^(^ ^1^ ^)^ It was estimated that 97 million children and adolescents under 15 years old were infected by BMT, that is, had a latent tuberculosis infection (LTBI).^(^ ^2^ ^)^

Brazil ranks 18^th^ in the number of TB cases, representing 0.9% of cases in the world and 33% of estimated cases in the Americas.^3^ In 2016, there were 66,796 new cases of TB and 12,809 cases of retreatment - the disease incidence coefficient was 32.4 cases per 100,000 inhabitants.^(^ ^4^ ^)^

The main measures for pulmonary TB control are active investigation, accurate diagnosis and immediate treatment. Another important strategy is to evaluate the patient's contacts to prevent the development of the disease by detecting and treating LTBI and diagnosing the active disease early on. ^(^ ^5^ ^)^

Diagnosing TB in children and adolescents is a challenge because sputum examinations usually show a low rate of positive results in this population (6.8%) in comparison to adults (52.0%).^(^ ^6^ ^)^ Moreover, immunodiagnostic tests cannot distinguish the active disease from LTBI,^(^ ^7^ ^)^ and the existing diagnostic scores and criteria present significant variation in sensitivity and specificity estimates because they were validated with different standards and populations.^(^ ^8^ ^)^

## OBJECTIVE

To describe the characteristics of patients with a diagnosis of tuberculosis and latent tuberculosis infection.

## METHODS

This is a retrospective study with data from the charts of patients diagnosed with TB and LTBI, who were evaluated in the outpatient clinic of ***Programa Einstein na Comunidade de Paraisópolis*** [Einstein Program in the Community of Paraisópolis] between 2012 and 2015. Tuberculosis was diagnosed when there was isolation of BMT in secretion or affected tissues, using the score system adopted by the Brazilian Ministry of Health,^(^ ^5^ ^)^ or when the imaging exams suggested the disease in symptomatic patients. Latent tuberculosis infection was diagnosed in asymptomatic patients who had been in contact with the disease and whose tuberculin skin test (TT) was positive (≥5mm in children aged 2 years or more or those with any immunosuppressive condition; and ≥10mm in children younger than 2 years of age). All children had received the BCG vaccine at birth. Latent tuberculosis infection was also diagnosed when the active disease was not confirmed in patients whose TT was positive, but with no index case identified.

The nutritional status was classified through the calculation of the body mass index (BMI), and BMI tables for age and sex from the World Health Organization (WHO). The underweight category included thinness and severe thinness. The overweight category included risk of overweight and overweight. The obese category included diagnoses of obesity and severe obesity.^(^ ^9^ ^)^

The variable “index case” was categorized as “yes” or “no” for each individual diagnosed with TB. Tables show only the absolute and relative frequency of cases categorized as “yes”. Probability distribution of the quantitative variables was verified by boxplots and the Shapiro-Wilk test. Qualitative variables were described as absolute and relative frequencies and quantitative variables were described as medians and quartiles (first and third quartiles), because the normal distribution of frequencies was not verified. To evaluate possible factors associated to TB and LTBI diagnoses and the homogeneity of measures between the sexes, we used χ^2^ or Fisher's exact tests for qualitative variables, and Mann-Whitney test for ordinal quantitative or qualitative variables, with a significance level of 5%. The study was approved by the Research Ethics Committee of ***Hospital Israelita Albert Einstein*** (CAAE: 53639016.6.0000.0071).

## RESULTS

A total of 77 patients were evaluated. Age varied between 6 months and 13.4 years. Most patients were male (54.5%), aged between 0 and 4 years (54.5%), with an LTBI diagnosis (64.9%), and categorized as eutrophic (71.2%). Of all patients, 24.7% were overweight or obese, fathers and mothers represented approximately 33% of index cases, and the index case was identified for 85.7% of patients. Two index cases from the same family (uncle and grandfather) were detected in two patients (Table 1).

**Table 1:** Characteristics of patients, distributed by sex

Variables		Sex		Total	p value
		Female	Male		
Age, years		4.6 [3.2-8.0]	4.3 [2.4-8.6]	4.4[2.8-8.3]	0.539*
Age group, years					0.833*
	0-4	18 (51.4)	24 (57.1)	42 (54.5)	
	5-9	12 (34.3)	10 (23.8)	22 (28.6)	
	≥10	5 (14.3)	8 (19.0)	13 (16.9)	
Diagnosis					0.191†
	LTBI	20 (57.1)	30(71.4)	50 (64.9)	
	TB	15(42.9)	12 (28.6)	27 (35.1)	
Index case					
	Grandparents	7(20.0)	3 (7.1)	10 (13.0)	0.171‡
	Parents	10 (28.6)	18 (42.9)	28 (36.4)	0.238‡
	Siblings	1 (2.9)	4(9.5)	5 (6.5)	0.369‡
	Uncles/aunties	6 (17.1)	6 (14.3)	12 (15.6)	0.762‡
	Others	5 (14.3)	8 (19.0)	13 (16.9)	0.803†
	Not identified	7(20.0)	4(9.5)	11 (14.3)	0.211‡
Nutritional status					0.161*
	Slimness	1 (3.0)	2 (5.0)	3 (4.1)	
	Eutrophy	27 (81.8)	25 (62.5)	52(71.2)	
	Overweight	4 (12.1)	10 (25.0)	14 (19.2)	
	Obesity	1 (3.0)	3(7.5)	4(5.5)	
Total		35 (45.5)	42 (54.5)	77 (100)	-

Age, in numerical format, is presented by median and [1^st^ – 3^rd^ quartilesj; other variables, by n (%).

* p values associated to Mann-Whitney test;

† χ^2^ test;

‡ Fisher's exact test.

LTBI; latent tuberculosis infection; TB: tuberculosis.

Tuberculin skin test was positive in 92% of individuals, and, in most cases, presented values >10mm (68.0%). All patients had a chest X-ray, and about three-quarters of those came back normal (72.7%). After the chest X-ray, computed tomography of the thorax (CTT) was the most requested exam - alone or together with other exams (29.9%) - followed by gastric aspirate examination (microscopy for bacilli and culture) (28.6%). Statistically significant differences were found between the sexes in the distribution of frequencies of other tests and BMT detection in the gastric aspirate (Table 2).

**Table 2:** Characteristics of patients’ exams, distributed by sex

Variables		Sex		Total	p value
		Female	Male		
TT value, mm		11.0 [7.3-16.0]	16.0 [9.0-20.0]	14.0 [8.0-19.5]	0.179*
TT range, mm					0.324*
	0-4	3(8.8)	3(7.3)	6(8.0)	
	5-9	10 (29.4)	8 (19.5)	18 (24.0)	
	>10	21 (61.8)	30 (73.2)	51 (68.0)	
TT					>0.999†
	Positive	31 (91.2)	38 (92.7)	69 (92.0)	
	Negative	3(8.8)	3(7.3)	6(8.0)	
Chest X-ray					0.207‡
	Altered	12 (34.3)	9 (21.4)	21 (27.3)	
	Normal	23 (65.7)	33(78.6)	56 (72.7)	
Performing other exams					0.278‡
	No	14 (40.0)	22(52.4)	36 (46.8)	
	Yes	21 (60.0)	20(47.6)	41 (53.2)	
Other exams					0.033†
	No	14 (40.0)	22(52.4)	36 (46.8)	
	BC + CTT	1 (2.9)	0 (0.0)	1 (1.3)	
	Lymph node biopsy	1 (2.9)	0 (0.0)	1 (1.3)	
	BMT in urine	0 (0.0)	1 (2.4)	1 (1.3)	
	BMT in GA	10 (28.6)	6 (14.3)	16 (20.8)	
	BMT in GA + CTT	5 (14.3)	1 (2.4)	6(7.8)	
	CTT	4(11.4)	12(28.6)	16 (20.8)	
Performing CTT					0.820‡
	No	25 (71.4)	29(69.0)	54 (70.1)	
	Yes	10 (28.6)	13 (31.0)	23 (29.9)	
BMT detection in GA					
	No	20 (57.1)	35 (83.3)	55 (71.4)	0.011‡
	Yes	15 (42.9)	7 (16.7)	22 (28.6)	
Total		35 (45.5)	42 (54.5)	77 (100)	–

TT, in numerical format, is presented by median and [1^st^ – 3^rd^ quartilesj; other variables, by n (%).

* p values associated to Mann-Whitney test;

† Fisher's exact test;

‡ χ^2^ test.

TT: tuberculin skin test; BC: bronchoscopy; CTT: computed tomography of thorax; BMT: detection and/or culture of *Mycobacterium tuberculosis* bacillum; GA: gastric aspirate.

The median age (5.0 years *vs.* 4.3 years) and the proportions of overweight and obesity (28.3% *versus* 18.5%) were higher in patients diagnosed with LTBI in comparison to those with TB, respectively. The proportion of non-identified index cases (22.2% *versus* 10.0%) was higher in patients with TB compared to those with LTBI, respectively. We did not detect, however, statistically significant differences in patients’ characteristic variables, when those were classified by diagnosis (Table 3).

**Table 3:** Characteristics of patients, distributed by diagnosis

Variables		Diagnosis		p value
		LTBI	TB	
Age, years		5.0 [2.9-8.8]	4.3 [2.1-7.1]	0.224*
Age groups, years				0.165*
	0-4	25(50.0)	17(63.0)	
	5-9	14 (28.0)	8 (29.6)	
	>10	11 (22.0)	2 (7.4)	
Index case				
	Grandparents	8 (16.0)	2 (7.4)	0.479†
	Parents	19 (38.0)	9 (33.3)	0.805†
	Siblings	3 (6.0)	2 (7.4)	>0.999†
	Uncles/aunties	10(20.0)	2(7.4)	0.197†
	Others	7 (14.0)	6 (22.2)	0.361†
	Not identified	5(10.0)	6 (22.2)	0.179†
Nutritional status				
	Slimness	1 (2.2)	2 (7.4)	0.186*
	Eutrophy	32 (69.6)	20 (74.1)	
	Overweight	9 (19.6)	5 (18.5)	
	Obesity	4 (8.7)	0 (0.0)	
Total		50 (64.9)	27 (35.1)	

Age, in numerical format, is presented by median and [1^st^ – 3^rd^ quartilesj; other variables, by n (%).

* p values associated to Mann-Whitney test;

† Fisher's exact test.

LTBI; latent tuberculosis infection; TB: tuberculosis.

We observed a significant difference between the proportions of alterations shown in chest X-ray, which was higher among TB cases (70.4%) in comparison to LTBI cases (4.0%). The first group also presented a higher proportion of performing CTT (55.6% *versus* 16.0%) and other exams (81.5% *versus* 38.0%) (Table 4).

**Table 4:** Characteristics of patients’ exams, distributed by diagnosis

Variables		Diagnosis		p value
		LTBI	TB	
TT value, mm		14.0 [9.0-19.0]	13.0 [5.8-20.0]	>0.999*
TT range, mm				
	0-4	0 (0.0)	6 (23.1)	0.630*
	5-9	16 (32.7)	2(7.7)	
	>10	33 (67.3)	18 (69.2)	
TT				0.001†
	Positive	49 (100.0)	20 (76.9)	
	Negative	0 (0.0)	6 (23.1)	
Chest X-ray				<0.001‡
	Altered	2 (4.0)	19 (70.4)	
	Normal	48 (96.0)	8(29.6)	
Performing other exams				<0.001‡
	No	31 (62.0)	5 (18.5)	
	Yes	19 (38.0)	22(81.5)	
Other exams				<0.001†
	No	31 (62.0)	5 (18.5)	
	BC + CTT	0 (0.0)	1 (3.7)	
	Lymph node biopsy	0 (0.0)	1 (3.7)	
	BMT in urine	1 (2.0)	0(0.0)	
	BMT in GA	10 (20.0)	6 (22.2)	
	BMT in GA + CTT	3(6.0)	3 (11.1)	
	CTT	5 (10.0)	11 (40.7)	
Performing CTT				<0.001‡
	No	42 (84.0)	12 (44.4)	
	Yes	8 (16.0)	15 (55.6)	
BMT detection in GA				0.497‡
	No	37 (74.0)	18 (66.7)	
	Yes	13 (26.0)	9(33.3)	
Total		50 (64.9)	27(35.1)	–

TT, in numerical format, is presented by median and [1^st^ – 3^rd^ quartiles]; other variables, by n (%).

* p values associated to Mann-Whitney test;

† χ^2^ test;

‡ Fisher's exact test.

LTBI: latent tuberculosis infection; TB: tuberculosis; TT: tuberculin skin test; BC: bronchoscopy; BMT: detection and/or culture of *Mycobacterium tuberculosis* bacillum; GA: gastric aspirate; CTT: computed tomography of thorax.

Of the patients with LTBI, two showed alterations in the chest X-ray and had a CTT. One of the patients’ CTT came back normal, and the other's showed alterations not consistent with TB. Of the eight patients with LTBI who had a CTT, two showed normal results, and six showed alterations not consistent with TB.

## DISCUSSION

Our study presented the characteristics of patients with diagnosis of tuberculosis and latent tuberculosis infection. Our sample profile was patients with a median age of 4.4 years, mostly male, eutrophic and with an LTBI diagnosis. To compare our results to the literature, we decided to evaluate studies done in outpatient settings, and with children and adolescents in Brazil. It is important, however, to consider that different dates, places, and evaluated samples may result in different characteristics of the patients.

We compared our results to those of a study conducted in the city of Jacarepaguá, in the Brazilian State of Rio de Janeiro between 2002 and 2006, which evaluated LTBI and TB patients aged <15 years old. The differences found in this comparison were as follows: regarding patients with LTBI, our study showed a similar proportion of normal chest X-rays (96.0% *versus* 100%) and a lower proportion of identified contact (90% *versus* 100%). In the comparison between TB patients, the median TT was similar (13mm *versus* 15mm), and the proportion of identified contact was lower (77.8% *versus* 95%).^(^ ^10^ ^)^ In our sample, approximately one third of index cases were identified as the patients’ mother or father, with no statistically significant differences between TB and LTBI patients.

When we compared our results to those of a study conducted in Salvador, in the State of Bahia, between 1997 and 2007, with TB patients <14 years old, we found a similar proportion of identified household contact (77.8% *versus* 80.4%), a higher proportion of normal chest X-rays (29.6% *versus* 0.3%), and a lower proportion of nutritional status adequacy (74.1% *versus* 82.2%).^(^ ^11^ ^)^ Regarding this last aspect, there is evidence that malnutrition is a risk factor for TB,^(^ ^12^ ^)^ and it is a score criterion for its diagnosis.^(^ ^5^ ^)^ However, in our sample, approximately 96% of patients diagnosed with TB were eutrophic or weight excess.

Comparing our results to those of a study from the city of Rio de Janeiro, State of Rio de Janeiro between 2002 and 2009, with patients aged <15 years diagnosed with LTBI, our study showed a larger proportion of male individuals (60.0% ***versus*** 51.4%), a smaller proportion of patients <10 years old (78.0% ***versus*** 86.7%), and a similar proportion of identified contacts (90.0% ***versus*** 92.3%).^(^ ^13^ ^)^

Regarding the tests used to diagnose the disease, a study with individuals aged <15 years presenting signs or symptoms suggestive of pulmonary TB showed different sensitivity results: 22% for direct microscopy, 60% for BMT culture, 64% for chest X-ray, and 75% for TT. For specificity, direct microscopy and BMT culture presented values close to 100%, chest X-ray was 78%, and TT was 69%.^(^ ^14^ ^)^

Children end up swallowing sputum when they cannot expectorate, and the gastric aspirate can be investigated for BMT. In this aspect, a literature review evaluated the accuracy of the gastric lavage/aspirate in children and adolescents under 15 years of age, and showed the exam has a low sensitivity for bacteriological confirmation, which is higher in BMT culture.^(^ ^15^ ^)^

Computed tomography of thorax which was evaluated using patients with bacterial pneumonia as a control group, showed some characteristics that may suggest the presence of TB in individuals <14 years old.^(^ ^16^ ^)^ For our patients who were symptomatic but had normal chest X-rays, performing CTT was essential to identify changes consistent with the presence or absence of TB.

Finally, we can use the risk score to diagnose TB in children.^(^ ^5^ ^)^ However, a study with patients under 15 years old showed different sensitivity and specificity, depending on the cut-off point used. For a score of 30, sensitivity and specificity were 78.6% and 69.2%, and a score of 40, they were 48.2% and 87.9%, respectively.^(^ ^17^ ^)^

Diagnosing TB in children is therefore challenging, especially considering the significant differences between individuals in this age group and adults, which go from the history of contact, risk factors, and diagnostic tests, to treatment and risk of transmission.^(^ ^18^ ^)^

The 2014 World Health Organization assembly approved a strategy called WHO End TB Strategy to reduce the incidence and overall mortality by TB in the next years.^(^ ^19^ ^)^ The number of children under 15 years old who live in households with adults with TB is estimated to be 7.5 million each year.^(^ ^20^ ^)^ This age group presents a higher risk of progressing to the disease^(^ ^18^ ^)^ and it is estimated that roughly 10% of them will become ill, mostly within one year of having been infected with BMT.^(^ ^21^ ^)^ Regarding treatment, we have been trying to identify the strategies that improve compliance^(^ ^22^ ^)^ to increase the chances of a cure and reduce resistance to medication^(^ ^23^ ^)^ and mortality by TB in children.^(^ ^24^ ^)^

Brazil has developed its national plan to eradicate TB as a public health problem and reduce its incidence and mortality by 2035. The plan defines the strategies to reach the established goals as three pillars: the first pillar is about prevention and integrated care centered on the individual; the second is about audacious policies and a support system; and the third addresses enhancing research studies and innovation.^(^ ^25^ ^)^ For this last pillar, our results can contribute with a better knowledge of the characteristics of TB and LTBI in children and adolescents and to help the search for the proposed objectives.

## CONCLUSION

In our sample, the proportions of chest X-rays with alterations, and performing computer tomography of the thorax and other exams in patients with a tuberculosis diagnosis were larger than in relation to the same proportions in patients with latent tuberculosis infection.
